# Mental health problems amongst school-age children and adolescents during the COVID-19 pandemic in the UK, Ireland and Iran: A call to action and research

**DOI:** 10.34172/hpp.2020.46

**Published:** 2020-11-07

**Authors:** Mohsen Rajabi

**Affiliations:** Department of Psychology, Faculty of Psychology and Educational Sciences, University of Tehran, Tehran, Iran

## Dear Editor,

To cope with the rapid spread of the coronavirus disease 2019 (COVID-19) outbreak, in 188 countries, governments have implemented swift, wide-ranging public health emergency measures that include partial or full lockdowns, school closures, social restrictions and self-isolating behaviours. According to the UNESCO monitoring, 107 countries imposed nationwide school closures and 65 countries implemented localised closures, impacting about 81.6% of the world’s student population at primary and secondary level.^1^ Although the scientific debate is ongoing about the effectiveness of school closures in controlling the virus transmission,^2^ long-term closures will have detrimental psychosocial and health consequences for children and adolescents around the world.

School shutdowns have significantly interrupted the academic life of many students and their families, which may bring about long-term effects on their social connectedness and mental health.^3^ Although there is ample evidence indicating the negative psychological impact of COVID-19 on the general population,^4^ young adults,^5^ and medical staff,^6^ not much is known about the effects on school-age children and adolescents. There is scant evidence and foundational research on the effects of home-quarantined students, particularly those with special educational needs or pre-existing mental health conditions. While many survey research studies have recently mentioned this important gap,^2^ there is still a lack of well-conducted research and prospective cohort studies at the national and global levels.^7^

Children and adolescents are generally considered to be healthy without any urgent need for regular medical check-ups and health care services.^8^ However, mental health services and educational support through schools are very important for pupils. Since a wide range of mental illnesses begin in childhood, and are diagnosable in the early stages, mental health services have been crucially important for the treatment of emotional and behavioural difficulties, especially in this strange time.^3^ If untreated, mental health problems amongst students can result in many detrimental health, social and learning consequences.

Schools provide many other essential services to students outside of education including school lunch, physical activity in the outdoor natural environment, and counselling sessions with professionals.^8^ A potentially overlooked role of schools is the delivery of health care, especially mental health services for students with special needs. Therefore, such school closures for children and adolescents mean a lack of access to the school-linked service. Students with special needs, such as those with neurodevelopmental and mental disorders, are also at high risk. For this group of vulnerable students, online education through television programmes or social media is not enough. While online learning might continue unimpeded for normal students, children and adolescents with emotional or behavioural difficulties are more likely to struggle to complete homework and online tutorials because of their precarious situations.^3^

In a cross-cultural survey by the Co-SPACE study,^9^ which included approximately 10 000 parents of children aged 4–18 years in the UK, Ireland and Iran, over 21.4% of the parents, on average, reported on a child with special educational needs, psychiatric and neurodevelopmental disorders (e.g. ADHD and ASD).^10,11^ Prior to the COVID-19 pandemic, around 44 percent of children with special educational needs, neurodevelopmental or mental health disorders were receiving different types of services from their schools. These supports are stopped or postponed due to the COVID-19 pandemic in the UK, Ireland and Iran; for example, about 60% of Irish and Iranian children and 80 percent of UK children with special educational needs or mental health problems, who were previously receiving such services, are no longer receiving this support.^10-12^ In the UK, parents of primary school-age children reported a statistically significant increase in the rate of emotional/behavioural problems and attentional difficulties in their children as lockdown progressed over a one-month period. However, secondary school-age children did not report any substantial change in their behavioural difficulties during the lockdown. Carers of children with special educational needs and those with a pre-existing mental disorder reported a reduction in their child’s emotional difficulties and no change in behavioural or attention difficulties.^10^

In conclusion, early findings from this study indicate that health authorities and governments should pay more attention to the potential associations that school closures and remote education have with children’s wellbeing, and make appropriate decisions to mitigate these issues for the upcoming academic year. As it seems that the COVID-19 pandemic is still ongoing, and future waves are likely to be worse than the spring/summer waves, it is crucial to investigate how prolonged school closures and virtual learning strategies can widen the gap between students with and without special educational needs or mental illness in the next academic year, particularly those from socially/economically disadvantaged backgrounds. Moreover, national legislators and policymakers should “monitor” the mental health of school-age students and try to deliver essential psychological and educational supports to close the current gap during the pandemic. To date, findings indicate that there are large gaps in the literature on the mental health consequences for children and youth during the COVID-19 pandemic, and that additional and more targeted research on child and adolescent mental health is needed.^7^ Without such actions, the existing situation may lead to a psychological and educational crisis amongst children and adolescents in the near future.

## Competing interests

The author declares that he has no competing interests.

## Ethical approval

Not applicable.
